# Antimicrobial and Biocide Resistance among Canine and Feline *Enterococcus faecalis*, *Enterococcus faecium*, *Escherichia coli*, *Pseudomonas aeruginosa*, and *Acinetobacter baumannii* Isolates from Diagnostic Submissions

**DOI:** 10.3390/antibiotics11020152

**Published:** 2022-01-25

**Authors:** Andrea T. Feßler, Anissa D. Scholtzek, Angela R. Schug, Barbara Kohn, Christiane Weingart, Dennis Hanke, Anne-Kathrin Schink, Astrid Bethe, Antina Lübke-Becker, Stefan Schwarz

**Affiliations:** 1 Institute of Microbiology and Epizootics, Centre for Infection Medicine, Department of Veterinary Medicine, Freie Universität Berlin, 14163 Berlin, Germany; anissa.scholtzek@bfr.bund.de (A.D.S.); a.schug@posteo.de (A.R.S.); dennis.hanke@fu-berlin.de (D.H.); anne-kathrin.schink@fu-berlin.de (A.-K.S.); astrid.bethe@fu-berlin.de (A.B.); antina.luebke-becker@fu-berlin.de (A.L.-B.); 2 Veterinary Centre for Resistance Research (TZR), Freie Universität Berlin, 14163 Berlin, Germany; barbara.kohn@fu-berlin.de (B.K.); christiane.weingart@fu-berlin.de (C.W.); 3 Unit Bacterial Toxins, Food Service, Department Biological Safety, German Federal Institute for Risk Assessment, 10589 Berlin, Germany; 4 Small Animal Clinic, Department of Veterinary Medicine, Freie Universität Berlin, 14163 Berlin, Germany

**Keywords:** dog, cat, infections, *E. faecalis*, *E. faecium*, *E. coli*, *P. aeruginosa*, *A. baumannii*, antimicrobial resistance (AMR), biocide susceptibility

## Abstract

A total of 215 isolates from infections of dogs and cats, including 49 *Enterococcus faecalis*, 37 *Enterococcus faecium*, 59 *Escherichia coli*, 56 *Pseudomonas aeruginosa*, and 14 *Acinetobacter baumannii*, were investigated for their susceptibility to 27 (Gram-positive bacteria) or 20 (Gram-negative bacteria) antimicrobial agents/combinations of antimicrobial agents by broth microdilution according to the recommendations of the Clinical and Laboratory Standards Institute. Moreover, all isolates were analysed for their susceptibility to the biocides benzalkonium chloride, chlorhexidine, polyhexanide, and octenidine by a recently published broth microdilution biocide susceptibility testing method. While the *E. faecalis* isolates did not show expanded resistances, considerable numbers of the *E. faecium* isolates were resistant to penicillins, macrolides, tetracyclines, and fluoroquinolones. Even a single vancomycin-resistant isolate that carried the *vanA* gene cluster was detected. Expanded multiresistance phenotypes were also detected among the *E. coli* isolates, including a single carbapenem-resistant, *bla*_OXA-48_-positive isolate. In addition, multiresistant *A. baumannii* isolates were detected. The minimal inhibitory concentrations of the biocides showed unimodal distributions but differed with respect to the biocide and the bacterial species investigated. Although there were no indications of a development of biocide resistance, some *P. aeruginosa* isolates exhibited benzalkonium MICs higher than the highest test concentration.

## 1. Introduction

Antimicrobial resistance (AMR) of bacteria is a major public health issue. The World Health Organization (WHO) recognized the importance of observing trends in and preventing the development of AMR in both veterinary and human medicine to tackle therapeutic challenges [1]. Of particular interest, therefore, are zoonotic pathogens, since transfer of (i) resistant bacterial isolates between humans and animals and/or (ii) resistance genes between the respective isolates are a matter of concern [2,3,4]. Occupational contact with livestock plays a major role in certain professions, such as veterinarians, slaughterhouse workers or farmers [5,6,7]. However, in the case of companion animals, such as dogs and cats, not only veterinarians but also animal owners are at risk of acquiring multiresistant zoonotic bacterial pathogens. Previous studies have shown that there are numerous examples of dog and cat owners sharing (multi)resistant bacteria with their pets [8,9,10,11]. Enterococci, in particular *Enterococcus faecalis* and *Enterococcus faecium*, but also the Gram-negative species *Escherichia coli*, *Pseudomonas aeruginosa*, and *Acinetobacter baumannii* have zoonotic potential. All of them cause infections in companion animals, especially skin, ear, or urinary tract infections [12,13,14,15,16,17]. In human medicine, some of these species, including *E. faecium*, *P. aeruginosa*, and *A. baumannii*, are among the so-called ESKAPE pathogens (which in addition to the aforementioned three pathogens also include *Staphylococcus aureus*, *Klebsiella pneumoniae*, and *Enterobacter* spp.) and play a relevant role as multiresistant pathogens in health care settings [18,19,20]. ESKAPE bacteria also occur in companion animals, such as dogs and cats, either as colonizers or causes of infections [21].

In addition to AMR, resistance to biocides is also an emerging issue [22]. A wide range of biocides of various classes, such as alcohols, bispyridines, aldehydes, biguanides, and quaternary ammonium compounds, are commonly used for disinfection purposes in households and clinical settings, including veterinary practices and clinics. Some biocides, e.g., chlorhexidine, have also been approved as components of shampoos to treat canine pyoderma [23,24,25]. The application of biocides also represents a selection pressure on bacteria prevalent in these settings or on these respective animals. As a consequence, these bacteria might also develop resistance to biocides. Acquired biocide resistance has been observed among *Serratia marcescens* (quaternary ammonium compounds with high level resistance), as well as *E. coli*, *Proteus mirabilis*, *P. aeruginosa*, and *S. marcescens* (chlorhexidine) [26,27]. Biocide-resistant strains have also been observed in healthcare settings, e.g., *Acinetobacter baumanii* isolates related to a nosocomial outbreak that required longer contact times with the biocide [28] or multiresistant *Klebsiella pneumoniae* isolates requiring an extended exposure time to chlorhexidine-based disinfectants [29]. In the present study, the susceptibility to benzalkonium chloride (a quaternary ammonium compound), chlorhexidine and polyhexanide (two biguanides), and octenidine (a bispyridine) was investigated. In contrast to antimicrobial susceptibility testing (AST) of bacteria, for which performance standards and quality control ranges for reference strains have been established by various institutions and organizations, such as the Clinical and Laboratory Standards Institute (CLSI), biocide susceptibility testing (BST) is still in its infancy. A harmonized protocol for BST via broth microdilution has recently been developed [30] and is followed in this study.

Our hypothesis was that multiresistant bacteria which may also exhibit reduced susceptibility to biocides occur in dogs and cats. Thus, the aim of the present study was to evaluate *E. faecalis*, *E. faecium*, *E. coli*, *P. aeruginosa*, and *A. baumannii* isolates from infections of cats and dogs for their susceptibility, not only to antimicrobial agents used for the control of infections caused by these bacterial pathogens, but also to four different widely used biocides. 

## 2. Results

### 2.1. Antimicrobial Susceptibility of Canine and Feline E. faecalis Isolates

The distribution of the minimal inhibitory concentration (MIC) data of the 35 canine and 14 feline *E. faecalis* isolates is shown in Table 1. All of the 49 *E. faecalis* isolates from infections of dogs and cats were classified as susceptible to penicillin G and ampicillin by applying the human-specific clinical breakpoints from CLSI document M100 [31], which have also been adopted in the CLSI document VET01S [32]. All isolates showed low MIC values for amoxicillin-clavulanic acid, which ranged between 0.06/0.03 and 1/0.5 mg/L (Table 1). Distinctly higher MICs were determined for all four cephalosporins tested (Table 1), which is in agreement with the fact that *E. faecalis* is considered as intrinsically resistant to cephalosporins [31].

Eighteen (36.7%) of the 49 isolates—ten from cats and eight from dogs—were tetracycline-resistant, with MICs ranging between 32 and 64 mg/L. All tetracycline-susceptible isolates were also doxycycline-susceptible [31], whereas all but one tetracycline-resistant isolates were doxycycline-resistant or -intermediate. A single canine tetracycline-resistant isolate showed a doxycycline MIC of 4 mg/L, which classified this isolate as borderline-susceptible. Erythromycin resistance was seen in four (8.2%) isolates—one from a cat and three from dogs. All erythromycin-resistant isolates also showed elevated MICs of tylosin (≥256 mg/L) and tilmicosin (64–≥256 mg/L). Thirteen feline and 25 canine *E. faecalis* isolates were classified as erythromycin-intermediate, while the remaining seven canine *E. faecalis* isolates were classified as erythromycin-susceptible. 

High-level gentamicin resistance (MIC > 500 mg/L) [31] was not observed. In contrast, four feline *E. faecalis* isolates exhibited high-level streptomycin resistance, with MICs of ≥1024 mg/L [31]. Resistance to ciprofloxacin was seen in three (6.1%) *E. faecalis* isolates, two from dogs and one from a cat [31]. One canine isolate was classified as borderline-resistant to ciprofloxacin and also showed lower MICs to enrofloxacin (1 mg/L) and marbofloxacin (2 mg/L) than the remaining two ciprofloxacin-resistant isolates (MICs 16 and ≥32 mg/L); their MICs to enrofloxacin and marbofloxacin were 16 and ≥32 mg/L or ≥32 mg/L, respectively. 

For linezolid, a single feline *E. faecalis* isolate was borderline-resistant (MIC 8 mg/L), two canine isolates were intermediate (MIC 4 mg/L), and all remaining isolates were susceptible (MICs 0.5–2 mg/L). All 49 canine and feline isolates were classified as vancomycin-susceptible (MICs 1–4 mg/L) and showed a unimodal MIC distribution for florfenicol. Although no breakpoints applicable to enterococci are available, isolates with the aforementioned low florfenicol MICs of 1–8 mg/L were tentatively considered as susceptible in accordance with previously published studies in which florfenicol-resistant enterococcal isolates showed distinctly higher MIC values [33,34].

Intrinsic resistance in *E. faecalis* is known for cephalosporins, clindamycin, aminoglycosides (except high-level resistance), nalidixic acid, tiamulin, trimethoprim-sulfamethoxazole, and quinupristin-dalfopristin. Despite the occurrence of low MICs—especially for trimethoprim-sulfamethoxazole—in single isolates of our data set, these isolates should not be reported as susceptible to the respective antimicrobial agents.

As intrinsic resistances are not considered for the calculation of single or multiple resistances [35], a total of 31/49 isolates were pansusceptible, i.e., susceptible to all antimicrobial agents tested; eight (three canine and five feline) isolates were resistant to only one class; eight (five canine and three feline) isolates to two classes; and only two feline isolates to three classes of antimicrobial agents. These latter two isolates (4.1%) were classified as multiresistant [35]. The multiple antimicrobial resistance (MAR) index of the *E. faecalis* isolates, calculated on the basis of resistances to penicillin G, erythromycin, ciprofloxacin, streptomycin (high level), gentamicin (high level), tetracycline, florfenicol, linezolid, and vancomycin, was 0.068.

### 2.2. Antimicrobial Susceptibility of Canine and Feline E. faecium Isolates

The distribution of the MIC data of the 27 canine and 10 feline *E. faecium* isolates is shown in Table 2. Among the 37 *E. faecium* isolates, 29 isolates (78.4%)—21 from dogs and eight from cats—showed resistance to penicillin G and 28 isolates (75.7%)—20 from dogs and eight from cats—to ampicillin [31]. A single canine isolate had a penicillin G MIC of 16 mg/L, which classified it as borderline resistant, and an ampicillin MIC of 4 mg/L, which classified this isolate as ampicillin-susceptible. The remaining 28 *E. faecium* isolates had high penicillin G MICs of ≥64 mg/L, while their ampicillin MICs ranged between 32 and ≥128 mg/L. All these isolates also exhibited elevated MICs to amoxicillin-clavulanic acid of at least 16/8 mg/L. The feline isolate IMT46708, sequenced because of its vancomycin resistance, was also resistant to ampicillin (MIC ≥128 mg/L) and exhibited 19 nucleotide substitutions in the gene *pbp5*, which codes for the low-affinity class B penicillin-binding protein 5 (PBP5). These substitutions resulted in the following amino acid exchanges: V24A, S27G, R34Q, G66E, A68T, E85D, E100Q, K144Q, T172A, L177I, D204G, A216S, T324A, M485A, N496K, A499T, E525D, E629V, and P667S. The simultaneous presence of these 19 amino acid substitutions has been described to be required for the expression of ampicillin resistance in *E. faecium* [36]. 

Twenty-seven (73.0%) of the 37 isolates—19 from dogs and eight from cats—were tetracycline-resistant [31]. Of them, 19 isolates—14 from dogs and five from cats—were also resistant to doxycycline. Six tetracycline-resistant isolates, three from dogs and cats each, were doxycycline-intermediate, while two canine tetracycline-resistant isolates were classified as doxycycline-susceptible. Another canine isolate was classified as tetracycline-intermediate (MIC 8 mg/L) and doxycycline-susceptible (MIC 2 mg/L). The sequenced isolate had tetracycline and doxycycline MICs of 128 mg/L and 16 mg/L and harbored the tetracycline resistance genes *tet*(L) and *tet*(M). Twenty-six isolates (70.3%)—18 from dogs and eight from cats—were erythromycin-resistant. Fifteen of them—13 from dogs and two from cats—showed high tylosin and tilmicosin MICs of ≥256 mg/L. The sequenced isolate had erythromycin, tylosin, and tilmicosin MICs of ≥64 mg/L, ≥256 mg/L, and ≥256 mg/L, respectively, and carried a constitutively expressed *erm*(B) gene as well as a *msr*(C) gene. 

High-level resistance to gentamicin (MIC >500 mg/L) was seen in 11 *E. faecium* isolates (29.7%)—nine from dogs and two from cats—while high-level resistance to streptomycin (MIC > 1000 mg/L) was encountered in 20 *E. faecium* isolates (54.1%)—17 from dogs and three from cats. The sequenced isolate had a streptomycin MIC of ≥1024 mg/L and carried the streptomycin resistance gene *aadE*. All 37 *E. faecium* isolates were resistant to ciprofloxacin, with MICs ranging between 4 and ≥32 mg/L. Their MICs of enrofloxacin and marbofloxacin were in the same range. The sequenced isolate exhibited MICs of ≥32 mg/L to all three tested fluoroquinolones and revealed a nucleotide exchange in the quinolone resistance determining region (QRDR) of *gyrA*, which resulted in the amino acid exchange S83I in the GyrA protein. Another nucleotide exchange was detected in the QRDR of *parC*, which led to the amino acid exchange S80R in the ParC protein. The simultaneous occurrence of both mutations has been described to be responsible for fluoroquinolone resistance [37]. For tiamulin, 24 isolates (64.9%)—21 from dogs and three from cats—exhibited high MICs of ≥64 mg/L, while the remaining 13 isolates had distinctly lower MICs in the range of 0.5–4 mg/L. Eighteen *E. faecium* isolates (48.6%)—16 from dogs and two from cats—were classified as borderline-resistant to quinupristin-dalfopristin (MIC 4–8 mg/L), and another five isolates—four from dogs and one from a cat—were classified as intermediate [31]. 

A single canine isolate (2.7%) exhibited a high florfenicol MIC of 32 mg/L, whereas eight canine isolates (21.6%) were considered linezolid-intermediate. Solely the sequenced feline isolate was vancomycin-resistant, with a MIC of ≥64 mg/L, and carried the *vanA* gene cluster composed of the genes *vanRSHAXYZ*.

*E. faecium* is considered as intrinsically resistant to cephalosporins, clindamycin, aminoglycosides (except high-level resistance), nalidixic acid, and trimethoprim-sulfamethoxazole. None of the 37 *E. faecium* isolates were pansusceptible. Instead, three (one canine and two feline) isolates were resistant to only one class, a single canine isolate to two classes, and the remaining 33 isolates to at least three classes of antimicrobial agents. These latter isolates (89.2%) were classified as multiresistant [35]. The MAR index of the *E. faecium* isolates, calculated on the basis of resistances to penicillin G, erythromycin, ciprofloxacin, streptomycin (high level), gentamicin (high level), tetracycline, tiamulin, florfenicol, linezolid, quinupristin-dalfopristin, and vancomycin, was 0.479.

### 2.3. Antimicrobial Susceptibility of Canine and Feline E. coli Isolates

The distribution of the MIC data of the 59 *E. coli* isolates—36 from dogs and 23 from cats—is shown in Table 3. *E. coli* is considered as intrinsically resistant to penicillin G but not to other β-lactams. Ampicillin and amoxicillin-clavulanic acid resistance were evaluated differentially for isolates from urinary tract infections and infections of other organ systems, as different body site-specific clinical breakpoints must be applied. In total, 17 isolates (28.8%), including three canine and 14 feline isolates, originated from urinary tract infections, and among them, two canine and four feline isolates were resistant to ampicillin when applying the dog-specific clinical breakpoints, as no cat-specific clinical breakpoints for ampicillin and feline *E. coli* isolates from urinary tract infections are available. This application of dog-specific breakpoints to feline isolates is in agreement with the CLSI document VET09 [38]. Only four of the six ampicillin-non-susceptible isolates from urinary tract infections (two feline and two canine isolates) were considered as non-susceptible to amoxicillin-clavulanic acid. The category “non-susceptible” is applicable when only clinical breakpoints for the category susceptible are available. For all isolates from disease conditions other than urinary tract infections (*n* = 42; 71.2%), the clinical breakpoints for skin and soft tissue infections of dogs and cats were applied. Using these clinical breakpoints, 41 isolates—33 from dogs and eight from cats—were resistant to ampicillin, while a single feline isolate was classified as ampicillin-intermediate. All 33 *E. coli* isolates from dogs and all nine *E. coli* isolates from cats proved to be resistant to amoxicillin-clavulanic acid. With regard to cephalosporins, 24 isolates (40.7%)—17 from dogs and seven from cats—were classified as resistant to cefotaxime using the human-specific clinical breakpoints from the CLSI document M100 [31]. A single canine isolate (1.7%) was borderline-resistant to imipenem with an MIC of 4 mg/L [31] and resistant to ampicillin (MIC ≥ 128 mg/L), amoxicillin-clavulanic acid (MIC ≥128/64 mg/L), and cefotaxime (MIC ≥64 mg/L). This isolate (IMT44896) was one of the three sequenced canine *E. coli* isolates, and sequence analysis revealed the presence of the oxacillinase gene *bla*_OXA-1_, the extended-spectrum β-lactamase (ESBL) gene *bla*_CTX-M-15_, and the carbapenemase gene *bla*_OXA-48_. The other two sequenced isolates (IMT41478 and IMT45150) were also resistant to the tested penicillins and cephalosporins with similarly high MICs. Their sequence analyses identified the broad-spectrum penicillinase gene *bla*_TEM-1B_, the ESBL gene *bla*_CTX-M-55_, and the AmpC β-lactamase gene *bla*_CMY-2_ (IMT41478), as well as the broad-spectrum penicillinase genes *bla*_TEM-1B_ and *bla*_TEM-135_, the ESBL genes *bla*_TEM-106_ and *bla*_TEM-126_, the oxacillinase gene *bla*_OXA-1,_ and the AmpC β-lactamase gene *bla*_CMY-2_ (IMT45150).

Twenty-five (42.4%) of the 59 isolates—21 from dogs and four from cats—were tetracycline-resistant [31]. Among them, 21 isolates were also classified as doxycycline-resistant [31]. The three sequenced isolates were tetracycline- and doxycycline-resistant and harbored the tetracycline resistance genes *tet*(A) (IMT41478, IMT44896) or *tet*(B) (IMT45150). Resistance to gentamicin was seen in 11 *E. coli* isolates—10 from dogs and one from a cat. Two of the sequenced isolates were gentamicin-resistant and harbored the gentamicin resistance genes *aac(3)-IIa* (IMT41478) or *aac(3)-IId* (IMT45150). Two of the sequenced isolates shared the highest streptomycin MIC measured and carried the streptomycin resistance genes *aph(3″)-Ib* (also known as *strA*), *aph(6)-Id* (also known as *strB*), and *ant(3″)-Ia* (also known as *aadA1*) (IMT41478, IMT45150) as well as *aadA2* (IMT45150). The third sequenced isolate had a streptomycin MIC of 16 mg/L and harbored the streptomycin resistance gene *aadA5*. The sequenced isolate IMT41478 had a neomycin MIC of 32 mg/L and carried the neomycin resistance gene *aph(3′)-Ia* (also known as *aphA1*). 

Ciprofloxacin resistance was detected in 23 isolates (39.0%)—17 from dogs and six from cats. These 23 isolates were also classified as marbofloxacin-resistant. Twenty-two of these isolates, with MICs of 16–≥32 mg/L, showed enrofloxacin resistance, while the remaining cipro- and marbofloxacin-resistant isolate was classified as enrofloxacin-susceptible, with a MIC of 0.5 mg/L. Two canine isolates were ciprofloxacin- and enrofloxacin-intermediate but susceptible to marbofloxacin. All fluoroquinolone-resistant isolates also shared high nalidixic acid MICs of ≥256 mg/L. Two of the sequenced isolates were ciprofloxacin-resistant and had a double mutation in *gyrA*, which led to the amino acid exchanges S83L and D87Y (IMT41478) or S83L and D87N (IMT45150) as well as single mutations in *parC* and *parE* that resulted in the amino acid exchanges S80I and S458A, respectively (IMT41478, IMT45150). In addition, isolate IMT45150 also had the mobile (fluoro)quinolone resistance gene *aac(6’)-Ib-cr*.

Resistance to trimethoprim-sulfamethoxazole was detected in 24 isolates (40.7%)—19 from dogs and five from cats. The three sequenced isolates were resistant, with MICs of 32/608 or ≥64/1216 mg/L, and harbored the sulphonamide resistance genes *sul2* and *sul3* (IMT41478), *sul1* (IMT44896), and *sul1* and *sul2* (IMT45150), as well as the trimethoprim resistance genes *dfrA14* (IMT41478), *dfrA17* (IMT44896), and *dfrA1*, *dfrA12*, and *dfrA36* (IMT45150). Two isolates with the highest florfenicol MICs of 256 and ≥512 mg/L were among the sequenced isolates (IMT41478, IMT45150), and both shared the florfenicol exporter gene *floR*. All 59 *E. coli* isolates were classified as colistin-intermediate.

Thus, none of the 59 *E. coli* isolates could be classified as pansusceptible, as at least an intermediate status for colistin was determined. Eighteen isolates—12 from dogs and six from cats—were resistant to one class, a single feline and three canine isolates to two classes, and the remaining 26 isolates—20 from dogs and six from cats—to at least three classes of antimicrobial agents. These latter isolates (44.1%) were classified as multiresistant [35]. The MAR index of the *E. coli* isolates, calculated on the basis of resistances to ampicillin, ciprofloxacin, trimethoprim-sulfamethoxazole, gentamicin, tetracycline, and colistin, was 0.398.

### 2.4. Antimicrobial Susceptibility of Canine and Feline P. aeruginosa Isolates

The distribution of the MIC data of the 56 *P. aeruginosa* isolates—53 from dogs and three from cats—is shown in Table 4. According to the CLSI documents M100 [31] and VET01S [32], *P. aeruginosa* is considered as intrinsically resistant to a number of antimicrobial agents including penicillin G, ampicillin, amoxicillin-clavulanic acid, cephalothin, cefotaxime, tetracycline, doxycycline, and trimethoprim-sulfamethoxazole. These intrinsic resistance properties correlate nicely with the corresponding high MIC values (Table 4). Three isolates with an imipenem MIC of 8 mg/L were considered as borderline imipenem-resistant. 

*P. aeruginosa* is also intrinsically resistant to chloramphenicol. Although only florfenicol has been tested in this study, the high florfenicol MICs (32–≥512 mg/L) suggest that the *P. aeruginosa* isolates tested in this study were also resistant to florfenicol. The use of the clinical breakpoints for gentamicin applicable to dogs [32] identified five isolates (8.9%), all of canine origin, as resistant to gentamicin. The streptomycin MICs showed a unimodal distribution, with the highest MIC values of 512 and ≥1024 mg/L being detected in gentamicin-resistant (*n* = 3) and gentamicin-intermediate (*n* = 1) isolates. Moreover, the neomycin MICs also showed a unimodal distribution.

Resistance to ciprofloxacin was seen in nine isolates (16.1%), all from dogs [31], that were also resistant to enrofloxacin when applying the cat-specific clinical breakpoints [32] and showed high marbofloxacin MICs of 4 to ≥32 mg/L. In addition, the single ciprofloxacin-intermediate isolate was resistant to enrofloxacin (4 mg/L) and had an elevated marbofloxacin MIC of 4 mg/L. One enrofloxacin-resistant isolate (4 mg/L) was classified as ciprofloxacin-susceptible, with a MIC of 0.5 mg/L. All ciprofloxacin- and/or enrofloxacin-resistant *P. aeruginosa* isolates exhibited high nalidixic acid MICs of ≥256 mg/L. For colistin, all isolates were classified as intermediate [31]. 

None of the 56 *P. aeruginosa* isolates were considered as pansusceptible, as for colistin, no susceptible category is defined, and thus, all isolates were at least intermediate to colistin. Since clinical breakpoints are only available for members of four classes of antimicrobial agents, 41 isolates did not exhibit resistance to any of these classes. Instead, 11 isolates exhibited resistance to one class and four isolates to two classes of antimicrobial agents. None of the isolates showed resistance to three or more classes of antimicrobial agents. Consequently, no isolate was classified as multiresistant, following published definitions [35]. The MAR index of the *P. aeruginosa* isolates, calculated on the basis of resistances to imipenem, enrofloxacin, gentamicin, and colistin, was 0.085.

### 2.5. Antimicrobial Susceptibility of Canine and Feline A. baumannii Isolates

The distribution of the MIC data of the 14 *A. baumannii* isolates—ten from dogs and four from cats—is shown in Table 5. According to the CLSI document M100 [31], *A. baumannii* is considered as intrinsically resistant to penicillin G, ampicillin, amoxicillin-clavulanic acid, and cephalothin. For cefotaxime, four isolates—one from a dog and three from cats—had a MIC of ≥64 mg/L and were classified as cefotaxime-resistant. The MICs of the remaining cephalosporins were elevated. Resistance to imipenem was not detected [31]. Similar to *P. aeruginosa*, *A. baumannii* is also intrinsically resistant to chloramphenicol. The high florfenicol MICs (32–256 mg/L) of the *A. baumannii* isolates in this study suggest that the respective isolates were resistant to florfenicol. Three isolates (21.4%)—one from a dog and two from cats—were classified as gentamicin-resistant. Although no clinical breakpoints applicable to *A. baumannii* isolates are currently available, the MIC distributions for streptomycin and neomycin were also bimodal, suggesting that there might be resistant subpopulations. 

Resistance to ciprofloxacin was detected in five isolates (35.7%)—one from a dog and four from cats. These five isolates also showed high MICs of enrofloxacin and marbofloxacin of 8 or 16 mg/L. Moreover, these five isolates also exhibited the highest nalidixic acid MICs of ≥256 mg/L. Four isolates (28.6%)—one from a dog and three from cats—proved to be trimethoprim-sulfamethoxazole-resistant. Three isolates (21.4%)—one from a dog and two from cats—were tetracycline- and doxycycline-resistant. All 14 *A. baumannii* isolates were considered as colistin-intermediate [31].

As a consequence, none of the 14 *A. baumannii* isolates were considered as pansusceptible, as all isolates were at least intermediate to colistin. In addition to colistin, CLSI-approved clinical breakpoints applicable to *A. baumannii* are only available for seven members of six classes of antimicrobial agents. Nine isolates—all from dogs—did not exhibit resistance to any of these classes. Two isolates exhibited resistance to two classes of antimicrobial agents, and three isolates (21.4%) were classified as multiresistant by showing resistance to five classes of antimicrobial agents. The MAR index of the *A. baumannii* isolates, calculated on the basis of resistances to cefotaxime, ciprofloxacin, trimethoprim-sulfamethoxazole, gentamicin, tetracycline, and colistin, was 0.226.

### 2.6. Biocide Susceptibility of Feline and Canine E. faecalis, E. faecium, E. coli, P. aeruginosa and A. baumannii Isolates

**Figure 1:**
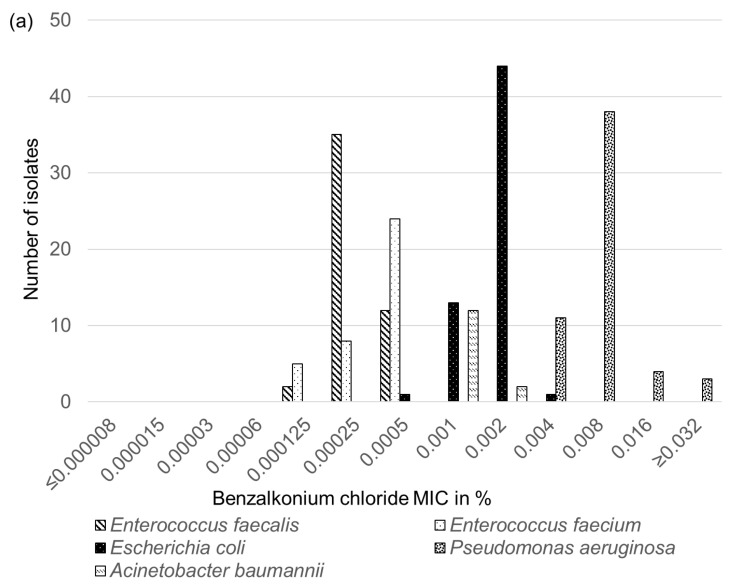

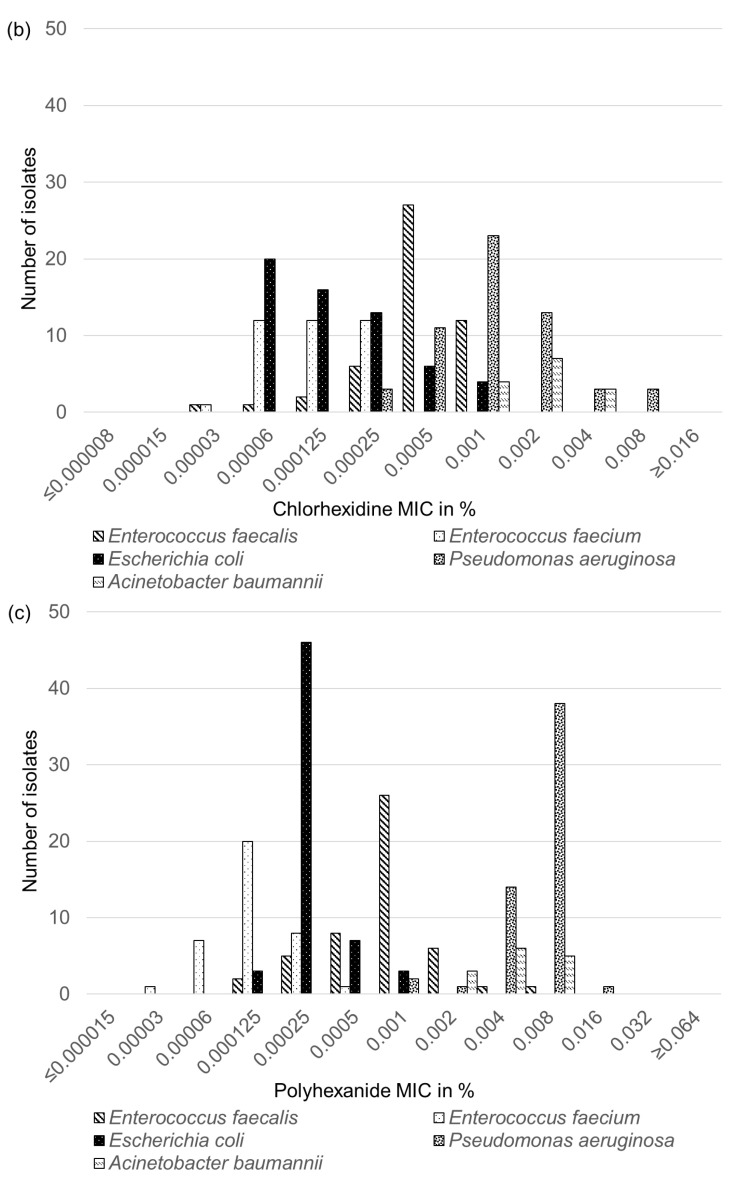

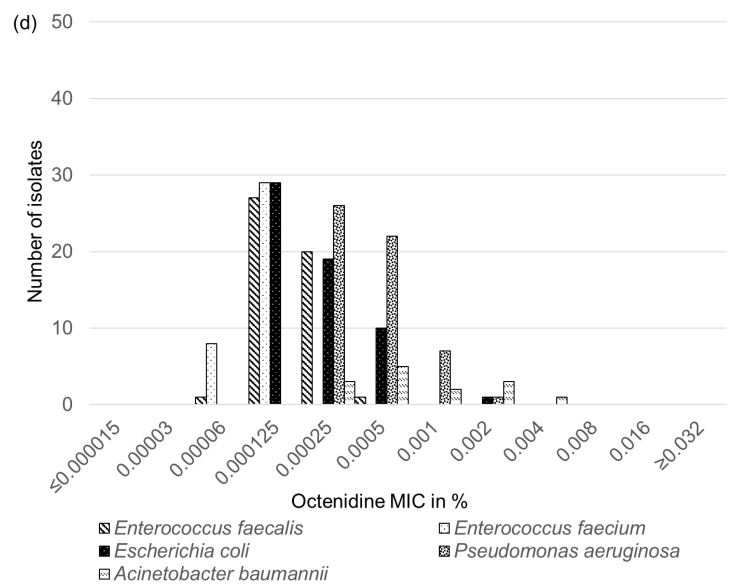
Distributions of the MIC values of the 49 *E. faecalis*, 37 *E. faecium*, 59 *E. coli*, 56 *P. aeruginosa*, and 14 *A. baumannii* isolates for the four biocides tested: (**a**) benzalkonium chloride, (**b**) chlorhexidine, (**c**) polyhexanide, and (**d**) octenidine.

Analysis of the 49 *E. faecalis*, 37 *E. faecium*, 59 *E. coli*, 56 *P. aeruginosa*, and 14 *A. baumannii* isolates for their susceptibility to the four biocides benzalkonium chloride, chlorhexidine, polyhexanide, and octenidine yielded unimodal distributions of the MICs for each biocide and bacterial species. As no differences in the MICs for the respective biocide were seen between the canine and feline isolates of each bacterial species, the corresponding canine and feline isolates are not further differentiated in Figure 1a–d. 

For benzalkonium chloride, the *E. faecalis* and *E. faecium* isolates exhibited MICs in the same range between 0.000125 and 0.0005%. Nevertheless, both species differed in the most frequently determined MIC, which was 0.00025% in 35 of the 49 *E. faecalis* isolates and 0.0005% in 24 of the 37 *E. faecium* isolates. In contrast, analysis of the *E. coli* isolates revealed higher benzalkonium MICs in the range between 0.0005 and 0.004%, with the majority of the isolates (*n* = 44/59) showing a MIC of 0.002%. The benzalkonium MICs of the *A. baumannii* isolates were in a similar range of 0.001 or 0.002% as the MICs of the *E. coli* isolates. The *P. aeruginosa* isolates were the least susceptible population. They exhibited the highest benzalkonium MICs, which varied between 0.004 and ≥0.32%. While the most frequently seen MIC in 38 of the 56 isolates was 0.008%, three *P. aeruginosa* isolates grew in the highest test concentration and exhibited MICs of ≥0.32% (Figure 1a).

For chlorhexidine, the MIC distributions of all tested bacterial species included three to six dilution steps. The *E. faecalis* isolates showed chlorhexidine MICs between 0.00003 and 0.001%, with the most frequently measured MIC in 27 of the 49 isolates at 0.0005%. In contrast, the MICs of the *E. faecium* isolates were in a lower range, between 0.00003 and 0.00025%, and more evenly distributed. Twelve isolates each exhibited chlorhexidine MICs of 0.00006, 0.000125, and 0.00025%. The chlorhexidine MICs of the *E. coli* isolates were in a similar range between 0.00006 and 0.001% as those of the *E. faecalis* isolates. However, the most frequently measured MIC in 20 of the 59 isolates was at the lower end of the distribution at 0.00006%. The *A. baumanni* isolates were less susceptible to chlorhexidine and showed a MIC distribution over three dilution steps between 0.001 and 0.004%, with half of the isolates (*n* = 7) having a MIC of 0.002%. The *P. aeruginosa* isolates exhibited chlorhexidine MICs between 0.00025 and 0.008%. The most frequently measured MIC in 23 of the 56 isolates was at 0.001%. Again, three *P. aeruginosa* isolates showed the highest chlorhexidine MIC measured in this study, at 0.008% (Figure 1b).

For polyhexanide, the *E. faecium* isolates showed the lowest MICs in a range between 0.00003 and 0.0005%, followed by the *E. coli* isolates in a slightly higher range of 0.000125 to 0.001%. The most frequently measured MIC of the *E. faecium* isolates was at 0.000125% in 20 of the 37 isolates, while that of the *E. coli* isolates was one dilution step higher at 0.00025% in 46 of the 59 isolates. The polyhexanide MICs of the *E. faecalis* isolates varied over seven dilution steps between 0.000125 and 0.008%, with the most frequently measured MIC at 0.001% in 26 of the 49 isolates. The MICs of the *A. baumannii* isolates were at the upper end of the aforementioned distribution and ranged between 0.002 and 0.008%. The *P. aeruginosa* isolates varied in their polyhexanide MICs between 0.001 and 0.016%, with the majority of the isolates (*n* = 38/56) having a MIC of 0.008%. Only a single *P. aeruginosa* isolate with a MIC of 0.016% was detected (Figure 1c). 

For octenidine, the *E. faecium* isolates showed the lowest MICs of 0.00006 (*n* = 8) or 0.000125% (*n* = 29), followed by the *E. faecalis* isolates. Their MIC range included four dilution steps between 0.00006 and 0.0005%. However, it should be noted that only single *E. faecalis* isolates exhibited the lowest and highest MIC values, while the majority of the isolates (*n* = 27/49) had a MIC of 0.000125%. The octenidine MICs of the *E. coli* isolates varied between 0.000125 and 0.002%, with 29 of the 59 isolates sharing a MIC of 0.000125%. The *P. aeruginosa* isolates were less susceptible to octenidine, as revealed by their MICs of 0.00025 to 0.002%. However, most of the isolates had MICs at the lower end of the distribution, i.e., 0.00025 (*n* = 26/56) and 0.0005% (*n* = 22/56). The *A. baumannii* isolates ranged in their MICs between 0.00025 and 0.004%. Only single isolates of *E. coli*, *P. aeruginosa,* and *A. baumannii* showed the highest octenidine MICs (Figure 1d). 

## 3. Discussion

AST as conducted in diagnostic laboratories provides helpful information to clinicians and veterinary practitioners for the choice of the most suitable antimicrobial agents. For veterinary applications of antimicrobial agents, veterinary-specific clinical breakpoints should be applied [35]. To achieve the best correlation between in vitro susceptibility and in vivo efficacy of an antimicrobial agent, clinical breakpoints applicable to the respective animal species and infected organ system have to be used. The CLSI document VET01S contains veterinary-specific clinical breakpoints for several antimicrobial agents applicable to *E. coli* and *P. aeruginosa* from dogs and cats [32]. Nevertheless, for certain antimicrobial agents tested in this study, such as ciprofloxacin, linezolid, vancomycin, or quinupristin-dalfopristin, no dog- or cat-specific clinical breakpoints are available. Hence, human-specific clinical breakpoints from the CLSI document M100 [31] have been applied to interpret the AST results. Moreover, all clinical breakpoints applicable to *E. faecalis* and *E. faecium* isolates, but also to *A. baumannii* isolates, are human-specific clinical breakpoints [31]. However, humans and companion animals, such as dogs and cats, do not share the same physiology, and the determination of clinical breakpoints takes into account the pharmacology of antimicrobial agents in the host organisms, the route of administration, and the dosage regimen, among other factors [35]. Thus, AST results of the aforementioned bacteria from dogs and cats interpreted by using human-specific clinical breakpoints should be considered with caution [38]. The same is true when using breakpoints applicable to bacteria from dogs for interpreting AST results of bacteria from cats and vice versa [38]. In general, it is noteworthy that in vitro activity of an antimicrobial agent, as shown by in vitro susceptibility of the causative bacteria, does not necessarily also mean in vivo efficacy. There are numerous factors, such as physicochemical conditions (oxygen partial pressure, perfusion rate, and pH value at the site of infection), biofilm-forming bacteria, or persisters, which may have an impact on the outcome of an antimicrobial therapy even if an antibacterial drug with suitable pharmacokinetic properties and an appropriate pharmaceutical formulation has been chosen and the in vitro susceptibility of the causative bacteria to this antimicrobial agent has been confirmed [39].

Multiresistance of bacterial pathogens is a problem in human and veterinary medicine. It is defined as resistance to at least three classes of antimicrobial agents [35], while definitions for extensive drug resistance and pandrug resistance to clinically significant livestock and companion animal bacterial pathogens have also been published [40]. Among the Gram-positive and Gram-negative bacteria investigated in this study, a considerable number of intrinsic resistances are known to be present. Since only acquired resistances are relevant for the calculation of multiresistance, the large number of intrinsic resistances, present in members of the five groups of bacteria, was neglected. Moreover, the definition of resistance requires the presence of clinical breakpoints, which, however, were not available in all cases, where bacteria showed in part high MICs of certain antimicrobial agents, e.g., *E. faecium* and tiamulin or *P. aeruginosa* and streptomycin. As a consequence, the large number of intrinsic resistances in addition to missing clinical breakpoints might lead to statements about the susceptibility status of at least some of the bacteria investigated in this study, which might appear to be misleading to readers who are not familiar with the determination of multiresistance and the calculation of MAR indices. When comparing the MAR indices determined in this study, *E. faecalis* and *P. aeruginosa*, both of which exhibit a considerable number of intrinsic resistances, had the lowest MAR indices of 0.068 and 0.085, respectively. In the case of *A. baumannii*, which also displays intrinsic resistance to a number of antimicrobial agents, the small number of isolates (*n* = 14) and the comparatively high number of (multi)resistant isolates (*n* = 5) on the one hand and the small number of antimicrobial classes for which clinical breakpoints were available (*n* = 6) on the other hand led to an elevated MAR index of 0.226. It needs to be mentioned that in small test populations of 10–20 isolates, few multiresistant isolates will have a disproportionately high influence on the MAR index. Hence, elevated MAR indices originating from small test populations should not be overestimated. Moreover, a low MAR index does not mean that the respective bacteria are susceptible to most antimicrobial agents. Instead, it means that the respective bacteria are susceptible to most antimicrobial agents for which applicable clinical breakpoints exist and that they might exhibit intrinsic resistances to a considerable number of antimicrobial agents approved for the respective animal species. 

When AST is conducted in routine diagnostics, the results that are communicated to clinicians and veterinary practitioners should clearly indicate to which antimicrobial agents intrinsic resistance is present, or the respective results should be set as “resistant” to avoid the use of these agents. This is of particular relevance for two reasons. First, the AST results of certain bacteria, might suggest that specific antimicrobial agents are active in vitro, although they are not effective clinically. For example, this is true for *Enterococcus* spp. and cephalosporins, aminoglycosides (except high level gentamicin and streptomycin resistance), clindamycin, and trimethoprim-sulfamethoxazole [31]. Second, persons involved in prescribing and applying antimicrobial agents in both human and veterinary medicine may lack in-depth knowledge of the broad field of intrinsic AMR. 

Only the single feline vancomycin-resistant *E. faecium* isolate and three canine *E. coli* isolates with unusually high florfenicol MICs—including one carbapenem-resistant isolate—have been subjected to whole genome sequencing. The *E. faecium* isolate was multiresistant and harbored a number of resistance genes previously detected in *Enterococcus* from companion animals [41,42,43]. Of particular importance was the observation that this isolate was co-resistant to ampicillin and vancomycin. A study from the Netherlands identified ampicillin-resistant *E. faecium* in dogs and cats as well as a single vancomycin-resistant *E. faecium* in a dog. However, an ampicillin- and vancomycin-co-resistant isolate was not detected among dogs or cats [44]. The first vancomycin-resistant *E. faecium* from a dog in Europe was described in 2009, although the genetic basis of resistance was not elucidated [45]. However, a canine vancomycin-resistant *E. faecium* isolate that harbored the *vanA* gene cluster was identified in 2002 in the USA [46]. 

The three *E. coli* isolates sequenced were also multiresistant. They all harbored a *floR* gene for combined resistance to chloramphenicol and florfenicol. Based on the very high florfenicol MIC values, we had suspected that at least a second gene that specifies a different resistance mechanism accounting for this resistance property was present. In *E. coli* from China, the multiresistance gene *cfr* had been detected occasionally together with the *floR* gene in *E. coli* isolates with high florfenicol MICs [47]. However, neither the gene *cfr* nor any other additional florfenicol resistance gene was detectable in the three *E. coli* isolates. The β-lactam, tetracycline, sulphonamide, trimethoprim, and aminoglycoside resistance genes, detected in the three *E. coli* isolates, represented resistance genes commonly found in *E. coli* from animal sources [48]. In contrast, the carbapenem resistance gene *bla*_OXA-48_ has rarely been identified in *E. coli* isolates from companion animals. There is a single report on canine *E. coli* from Germany, that described the occurrence of this carbapenemase gene on a plasmid-borne transposon [49]. The emergence of carbapenemase-producing bacteria in companion animals has been regarded as a public health concern because of the close contact between humans and their pets and the potential for cross-species transmission [50,51]. However, carbapenems are not licensed for use in animals worldwide, and hence a very low direct selection pressure exists under which bacteria of animal origin can acquire carbapenem resistance genes. Thus, other authors suggested that the real threat related to carbapenem resistance in humans does not come from animals, including companion animals, but from (i) the increased consumption of carbapenems in humans worldwide and (ii) the overall increase in human population movements worldwide, including migration and tourism [52]. 

There are no similar studies on the phenotypic AMR of canine and feline *E. faecalis*/*E. faecium* and *A. baumannii* isolates from Germany. However, at least one other study from Germany, the so-called BfT-GermVet study, which was conducted in 2004–2006, can be used for comparison of the resistance rates of the canine and feline *E. coli* and *P. aeruginosa* isolates, as the same CLSI methodology and quality control strains were used [53,54]. 

Ampicillin resistance was the most frequently detected resistance property in the BfT-GermVet study, with resistance rates of 39.3% recorded for 11/28 *E. coli* isolates from respiratory tract infections and 24.0% for 24/100 *E. coli* isolates from urinary/genital tract infections of dogs and cats [53]. In the present study, ampicillin resistance was also the dominant resistance property but with a distinctly higher resistance rate of 79.7% (47/59), which also included the six isolates from urinary tract infections classified as non-susceptible. In addition, the numbers of amoxicillin-clavulanic acid-resistant isolates varied distinctly between the two studies. While resistance to amoxicillin-clavulanic acid was rarely detected in the BfT-GermVet study, i.e., 3.6% (1/28) and 1.0% (1/100) in the two test populations, 78.0% (46/59) of the isolates in the current study (including the four isolates from urinary tract infections classified as non-susceptible) showed this resistance property. Tetracycline resistance (25/59, 42.4%) was the third most prevalent resistance property in the present study. This resistance rate was also substantially higher than the percentages of tetracycline-resistant *E. coli* in the BfT-GermVet study, namely 14.3% (4/28) and 15.0% (15/100) for the two groups of isolates. Resistance to trimethoprim-sulfamethoxazole was detected in 40.7% (24/59) of the isolates in the present study, whereas it was detected in 17.9% (5/28) and 11.0% (11/100) of *E. coli* isolates in the BfT-GermVet study. Enrofloxacin resistance was detected in 37.3% (22/59) of the isolates of the present study, while the *E. coli* isolates from the BfT-GermVet study exhibited enrofloxacin resistance at distinctly lower frequencies of 3.6% (1/28) and 1.0% (1/100) [53]. Gentamicin resistance was detected in 18.6% (11/59) of the *E. coli* isolates in the present study and in only 7.1% (2/28) and 3.0% (3/100) of the *E. coli* isolates in the BfT-Germ-Vet study. In summary, regardless of the AMR property investigated, the recent isolates showed distinctly higher percentages of resistance. The reasons for these increases in the resistance rates remain to be identified, especially since the sales figures of antimicrobial agents approved for use in animals have substantially declined since 2011 in Germany [55].

An opposite trend was seen for the *P. aeruginosa* isolates. The 71 isolates from the BfT-GermVet study [54] were distinctly less susceptible than the 56 isolates from the present study. Fluoroquinolone resistance declined from 23.9% (17/71) to 16.1% (9/56), and gentamicin resistance from 26.8% (19/71) to 8.9% (5/56). Re-evaluation of the colistin MICs revealed the presence of 5.6% (4/71) colistin-resistant isolates (MICs 4 or 8 mg/L) in the BfT-GermVet study, while all isolates of the present study were classified as intermediate, with MICs of ≤2 mg/L. Imipenem was not tested in the BfT-GermVet study.

The MICs determined for each of the four different biocides and the five different groups of bacteria showed unimodal distributions. In general, the Gram-negative non-fermenters *P. aeruginosa* and *A. baumannii* exhibited the highest MICs regardless of the biocide tested, whereas the Gram-positive *E. faecium* usually displayed the lowest MICs. No associations between resistance to specific antimicrobial agents and biocides in the same isolate could be observed. For benzalkonium chloride, a few *P. aeruginosa* isolates displayed MICs of ≥0.032%, which is at least one dilution step higher than the highest concentration tested. In these cases, it should be noted that benzalkonium chloride concentrations higher than 0.016% could not be prepared due to solubility problems. Isolates with these high benzalkonium MICs may be able to withstand the in-use concentration for disinfection of the skin of up to 0.01–0.2% [56]. To date, shampoos containing chlorhexidine and miconazole are approved for cats and dogs. The highest chlorhexidine MICs of 0.004% and 0.008%, determined for *P. aeruginosa* and *A. baumannii* isolates, were distinctly below the concentration of up to 4% in shampoos for veterinary applications [23,24]. No medicinal products that contain polyhexanide or octenidine are currently approved for veterinary use. So far, very few MIC data on the tested biocide/bacteria combinations are available for comparison. A study on five *mcr-1*-positive *E. coli* isolates from turkeys in Serbia identified MICs of 0.001 or 0.002% for benzalkonium chloride and 0.00003 or 0.00006% for chlorhexidine [57]. These benzalkonium MICs were also the two most frequently measured MICs in the present study. A considerable number of chlorhexidine MICs in the present study were higher than the aforementioned MICs, although a chlorhexidine MIC of 0.00006% was also the most frequently determined MIC in the present study. In another study on 104 porcine *E. coli* from Austria, the benzalkonium chloride MICs ranged from 0.0005 to 0.002%, with the majority of isolates displaying MICs of 0.001% (59/104, 54.7%) or 0.002% (43/104, 41.3%) [58]. This finding is in excellent agreement with the benzalkonium MICs determined for the canine and feline *E. coli* in the present study. The corresponding chlorhexidine MICs of the porcine *E. coli* ranged between 0.00003 and 0.002% [58]. The chlorhexidine MICs determined in the present study were in a similar range, although we could not detect *E. coli* isolates with the lowest and highest MICs of 0.00003% and 0.002%. In a third study, 27 *E. coli* isolates from marine mammals exhibited benzalkonium chloride MICs that ranged between 0.000125 and 0.002%, with most isolates (24/27) displaying a MIC of 0.002% [59]. The corresponding chlorhexidine MICs were distributed over six dilution steps from 0.0003% to 0.001% with the majority of isolates (24/27) ranging between 0.00006 and 0.0005% [59]. Again, these MICs were in an excellent agreement with the benzalkonium chloride and chlorhexidine MICs determined in the present study. These findings support the assumption that *E. coli* isolates regardless of their animal origin exhibit very similar MICs to the biocides benzalkonium chloride and chlorhexidine.

## 4. Materials and Methods

### 4.1. Origin and Identification of the Isolates Investigated

In total, 215 isolates, including 49 *E. faecalis* (35 from infections of dogs and 14 from infections of cats), 37 *E. faecium* (27 from infections of dogs and ten from infections of cats), 59 *E. coli* (36 from infections of dogs and 23 from infections of cats), 56 *P. aeruginosa* (53 from infections of dogs and three from infections of cats), and 14 *A. baumannii* (ten from infections of dogs and four from infections of cats), obtained from diagnostic submissions to the Institute of Microbiology and Epizootics, Centre for Infection Medicine, Department of Veterinary Medicine, Freie Universität Berlin, Berlin, Germany, were included in this study. The majority of the dogs and cats, from which the respective isolates originated, were presented with a wide range of infections at the Small Animal Clinic, Department of Veterinary Medicine, Freie Universität Berlin, Berlin, Germany, but also other veterinary clinics or practices in Germany between 01/2017 and 12/2019. The origin of the isolates is shown in Table 6. All *E. faecalis*, *E. faecium*, *E. coli*, *P. aeruginosa*, and *A. baumannii* isolates were from individual unrelated dogs and cats.

The bacterial isolates investigated in this study were identified by standard microbiological procedures as previously described [52,53,54,60,61,62]. In brief, the samples were analysed by aerobic cultivation after direct inoculation on suitable agar plates (all agar purchased from Oxoid, Wesel, Germany). For the detection of aerobic bacteria, Columbia blood agar (5% sheep blood), Gassner agar, and Brilliance UTI Clarity agar were inoculated within 12 h after sampling and incubated at 36 °C under aerobic conditions overnight. Except for the isolates from urinary tract infections, an additional enrichment in brain heart infusion broth (Oxoid) was performed for each sample at 37 °C for 18 h. Thereafter, an aliquot was transferred to Columbia blood agar (5% sheep blood). Species identification was based on colony morphology evaluation and verified by matrix-assisted laser desorption/ionization time-of-flight mass spectrometry with Bruker Microflex LT in combination with Flex Control (flexControl Version 3.4) and BIOTYPER (MBT Compass 4.1) software (Bruker Daltonics, Bremen, Germany).

### 4.2. Antimicrobial Susceptibility Testing

Minimal inhibitory concentrations (MICs) of the 215 isolates were determined using different panels of antimicrobial agents for the Gram-positive and the Gram-negative bacteria. All bacteria were tested for their susceptibility to penicillin G, ampicillin, amoxicillin-clavulanic acid, cephalothin, cefotaxime, cefoperazone, ceftiofur, florfenicol, streptomycin, neomycin, gentamicin, ciprofloxacin, enrofloxacin, marbofloxacin, nalidixic acid, trimethoprim-sulfamethoxazole, tetracycline, and doxycycline. Additional antimicrobial agents tested for *Enterococcus* spp. were erythromycin, tylosin, tilmicosin, linezolid, tiamulin, vancomycin, and quinupristin-dalfopristin (Table 1 and Table 2), and for Gram-negative bacteria imipenem and colistin (Table 3, Table 4 and Table 5). AST was conducted by broth microdilution according to CLSI recommendations [31,32]. In brief, a bacterial suspension, equivalent to a 0.5 McFarland Standard, was prepared by the colony suspension method. Five µL of the bacterial suspension were added per mL test medium, which was cation-adjusted MuellerHinton broth. The microtitre plates were inoculated with 50 µL inoculum per well and incubated for 16–20 h (*Enterococcus* spp., *E. coli* and *P. aeruginosa*) or 20–24 h (*A. baumannii*) at 35 °C ± 2 °C in ambient air. The results for vancomycin and *Enterococcus* spp. were read after incubation for 24 h under the aforementioned conditions. The microtitre plate layouts (Sensititre^®^) used in the national resistance monitoring program GE*RM*-Vet were also employed in this study. The antimicrobial agents tested and the test ranges in mg/L are displayed in Table 1, Table 2, Table 3, Table 4 and Table 5. *E. faecalis* ATCC^®^ 29212, *S. aureus* ATCC^®^ 29213, *E. coli* ATCC^®^ 25922 and *P. aeruginosa*^®^27853 served as quality control strains.

Dog- and cat-specific clinical breakpoints as listed in the CLSI document VET01S [32] were applied whenever possible. In the absence of cat-specific clinical breakpoints, dog-specific clinical breakpoints were occasionally used for feline isolates and vice versa. These extrapolations are in agreement with the recommendations given in the CLSI document VET09 [38]. In the absence of veterinary-specific clinical breakpoints for isolates from dogs and cats, human-specific clinical breakpoints from CLSI document M100 [31] were applied. 

### 4.3. Calculation of the MAR Index

The calculation of the MAR index followed the proposal of Krumperman [63] using the formula *a/(b* × *c)*, with *a* being the sum of all resistance properties observed in isolates of the respective bacterial species, e.g., *E. faecalis*, *b* is the number of antimicrobial agents tested and *c* is the number of all isolates of this bacterial species. To avoid bias by including several antimicrobial agents from the same class, only the class representatives or individual antimicrobial agents for which no cross-resistance is known were included in this calculation. Intrinsic resistance properties present in the different bacterial species were excluded from the calculation of the MAR indices. 

### 4.4. Whole Genome Sequencing and Analysis

Four isolates, one *E. faecium* and three *E. coli* isolates, were selected for whole genome sequence analysis based on particular rarely detected phenotypic resistance properties. The *E. faecium* isolate was resistant to vancomycin. The three *E. coli* isolates showed high florfenicol MICs, and one of them also resistance to imipenem. DNA was extracted using a QIAamp^®^ DNA Mini Kit (QIAGEN, Hilden, Germany). Libraries were prepared with the Nextera XT library preparation kit (Illumina Inc., San Diego, CA, USA). Using the Illumina MiSeq platform, a 2 × 300 bp paired-end sequencing in 30-fold multiplexes was performed. De novo assembling was carried out using Newbler (Roche, Basel, Switzerland) and SPAdes v3.12.0. The whole genome sequences were annotated with the subsystem technology server (RAST) and Prokka, which were checked with BLAST (National Center for Biotechnology Information, Rockville Pike, Bethesda, MD, USA) results. Using ResFinder from the Center for Genomic Epidemiology (https://genomicepidemiology.org, accessed on 1 December 2021), the sequences were searched for antimicrobial resistance genes and resistance-mediating mutations.

### 4.5. Biocide Susceptibility Testing

All 215 isolates were tested for their susceptibility to the following four biocides: benzalkonium chloride, chlorhexidine, octenidine dihydrochloride (octenidine), and polyhexamethylene biguanide hydrochloride (polyhexanide). BST was performed by broth microdilution according to a recently developed protocol [30]. This protocol was modified as follows. For the inoculum preparation, 30 µL of a bacterial suspension with a density of 0.5 McFarland were added to 12 mL tryptic soy broth (TSB). The microtitre plates were inoculated with 100 µL per well as recommended by the manufacturer of the custom-made microtitre plates (sifin diagnostics GmbH, Berlin, Germany). These plates contained the biocides in 11 or 12 two-fold dilution steps: benzalkonium chloride (0.000008–0.016%), chlorhexidine (0.000008–0.008%), octenidine (0.000016–0.016%), and polyhexanide (0.000016–0.032%). 

## 5. Conclusions

The results of this study show that enterococci, *E. coli* as well as the two non-fermenters *P. aeruginosa* and *A. baumannii* are involved in numerous infections of dogs and cats. Many of these canine and feline bacterial pathogens exhibited (multi)resistance to antimicrobial agents approved for use in dogs and cats. In addition, almost all of the bacterial species investigated showed intrinsic resistance to numerous antimicrobial agents. Both acquired and intrinsic resistance properties drastically reduce the options for antimicrobial therapy. Bearing this in mind, the performance of AST prior to the start of an antimicrobial therapy is of particular significance. As the results of AST are intended to guide the application of antimicrobial agents in small animal medicine, the correct interpretation and classification of bacteria as susceptible, intermediate, or resistant will help to avoid the use of antimicrobial agents to which the causative bacterial isolates show resistance under in vitro conditions. The substantial increase in resistances to virtually all classes of antimicrobial agents among *E. coli* isolates within a bit more than a decade underlines the need for prudent use of antimicrobial agents, in particular of those classified as critically important in human medicine. This is of outstanding relevance, as isolates displaying resistance to last-choice antimicrobial agents, such as a vancomycin-resistant *E. faecium* and a carbapenem-resistant *E. coli*, have been identified in this study. In contrast to the AST results, the BST results revealed unimodal MIC distributions, which did not suggest acquired biocide resistance development. However, for *P. aeruginosa* and benzalkonium chloride, the highest MICs measured were in the range of the in-use concentrations, suggesting a potential inefficacy of this biocide. Thus, BST, which is currently only rarely performed, will gain increasing relevance in the future. 

## Figures and Tables

**Table 1:** Minimal inhibitory concentrations of the 49 canine and feline *E. faecalis* isolates.

Antimicrobial Agent(s)	Number of Isolates for Which the MIC Value (mg/L) Is																	
	0.008	0.015	0.03	0.06	0.12	0.25	0.5	1	2	4	8	16	32	64	128	256	512	1024
Penicillin G		-	-	-	-	-	-	5	12	32	-	-	-	-				
Ampicillin			-	1	2	4	5	35	2	-	-	-	-	-	-			
Amoxicillin-clavulanic acid (2:1)			-	1	3	7	16	22	-	-	-	-	-	-	-			
Cephalothin				-	-	-	-	-	-	-	-	6	43	-	-	-		
Cefotaxime		-	-	-	1	-	-	1	-	-	-	-	-	47				
Cefoperazone				-	-	-	-	-	-	-	4	12	28	5				
Ceftiofur			-	-	1	-	1	1	-	-	1	1	6	15	23			
Florfenicol					-	-	-	2	20	26	1	-	-	-	-	-	-	
Erythromycin		-	-	-	-	4	3	6	20	12	-	-	-	4				
Tylosin				-	-	-	3	20	15	6	1	-	-	-	-	4		
Tilmicosin				-	-	-	-	-	-	1	1	28	13	3	-	3		
Clindamycin			-	-	1	-	-	-	1	-	2	30	12	1	2			
Streptomycin						-	-	-	-	-	-	-	-	6	31	8	-	4
Gentamicin					-	-	-	-	-	1	10	31	7	-	-	-	-	
Neomycin					-	-	-	-	-	-	1	-	1	8	39			
Ciprofloxacin	-	-	-	-	-	-	-	30	16	1	-	1	1					
Enrofloxacin	-	-	-	-	-	-	8	36	3	-	-	1	1					
Marbofloxacin	-	-	-	-	-	-	-	2	38	7	-	-	2					
Nalidixic acid				-	-	-	-	-	-	-	-	-	-	-	1	48		
Trimethoprim-sulfamethoxazole (1:19)		6	23	15	1	2	-	1	-	1	-	-	-	-				
Tetracycline					4	15	5	7	-	-	-	-	4	14	-	-	-	
Doxycycline				4	17	4	6	-	-	1	11	4	1	-	-	1		
Linezolid			-	-	-	-	1	15	30	2	1	-	-	-	-			
Tiamulin			-	-	-	-	-	1	-	-	-	1	-	-	47			
Vancomycin		-	-	-	-	-	-	31	15	3	-	-	-	-				
Quinupristin-dalfopristin		-	-	-	-	1	-	2	-	3	37	6	-	-				

The black areas are the test ranges not included in the test panels for the respective antimicrobial agents. Isolates that had no growth in any of the concentrations were given the lowest MIC value. Isolates with growth in all tested concentrations were given the next serially higher MIC value above the highest tested concentration (white number on black background). The MIC values of amoxicillin-clavulanic acid (2:1) and trimethoprim-sulfamethoxazole (1:19) are expressed as the MIC values of amoxicillin and trimethoprim, respectively. Different gray shading indicates the categories: susceptible—light gray; intermediate—middle gray; resistant—dark gray. For the clinical breakpoints used, please see Materials and Methods, Section 4.2.

**Table 2:** Minimal inhibitory concentrations of the 37 canine and feline *E. faecium* isolates.

Antimicrobial Agent (s)	Number of Isolates for Which the MIC Value (mg/L) Is																	
	0.008	0.015	0.03	0.06	0.12	0.25	0.5	1	2	4	8	16	32	64	128	256	512	1024
Penicillin G		-	-	-	-	-	1	-	4	3	-	1	-	28				
Ampicillin			-	-	1	-	1	4	2	1	-	-	1	9	18			
Amoxicillin-clavulanic acid (2:1)			-	-	1	2	2	3	1	-	-	1	9	2	16			
Cephalothin				-	-	-	-	-	-	-	1	2	2	2	1	29		
Cefotaxime		-	-	-	1	-	-	1	-	-	1	-	-	34				
Cefoperazone				-	-	-	-	-	-	-	2	1	3	31				
Ceftiofur			-	1	-	-	1	-	-	-	-	-	1	1	33			
Florfenicol					-	-	1	1	-	29	5	-	1	-	-	-	-	
Erythromycin		-	-	-	-	-	-	2	4	5	3	1	-	22				
Tylosin				-	-	1	7	9	1	2	2	-	-	-	-	15		
Tilmicosin				-	-	-	-	-	-	-	2	12	8	-	-	15		
Clindamycin			-	-	3	6	1	1	1	1	6	4	2	1	11			
Streptomycin						-	-	-	-	-	-	-	8	9	-	-	-	20
Gentamicin					-	-	-	-	-	4	17	4	-	1	-	-	11	-
Neomycin					-	-	-	-	1	3	5	11	3	1	13			
Ciprofloxacin	-	-	-	-	-	-	-	-	-	6	3	3	25					
Enrofloxacin	-	-	-	-	-	-	-	-	-	7	3	2	25					
Marbofloxacin	-	-	-	-	-	-	-	-	-	4	6	2	25					
Nalidixic acid				-	-	-	-	-	-	-	-	-	-	2	2	33		
Trimethoprim-sulfamethoxazole (1:19)		12	6	9	8	2	-	-	-	-	-	-	-	-				
Tetracycline					-	1	7	1	-	-	1	-	2	3	22	-	-	
Doxycycline				-	6	3	-	-	1	2	6	17	2	-	-	-		
Linezolid			-	-	-	-	2	1	26	8	-	-	-	-	-			
Tiamulin			-	-	-	-	2	8	1	2	-	-	-	1	23			
Vancomycin		-	-	-	-	-	19	11	5	1	-	-	-	1				
Quinupristin-dalfopristin		-	-	-	-	1	9	4	5	16	2	-	-	-				

The black areas are the test ranges not included in the test panels for the respective antimicrobial agents. Isolates that had no growth in any of the concentrations were given the lowest MIC value. Isolates with growth in all tested concentrations were given the next serially higher MIC value above the highest tested concentration (white number on black background). The MIC values of amoxicillin-clavulanic acid (2:1) and trimethoprim-sulfamethoxazole (1:19) are expressed as the MIC values of amoxicillin and trimethoprim, respectively. Different gray shading indicates the categories: susceptible—light gray; intermediate—middle gray; resistant—dark gray. For the clinical breakpoints used, please see Materials and Methods, Section 4.2.

**Table 3:** Minimal inhibitory concentrations of the 59 canine and feline *E. coli* isolates.

Antimicrobial Agent (s)	Number of Isolates for Which the MIC Value (mg/L) Is																	
	0.008	0.015	0.03	0.06	0.12	0.25	0.5	1	2	4	8	16	32	64	128	256	512	1024
Penicillin G		-	-	-	-	-	-	-	-	-	1	-	12	46				
Ampicillin			-	-	-	-	1	-	4	6	-	-	-	-	31			
Ampicillin (UTI)			-	-	-	-	-	-	7	4	-	-	-	-	6			
Amoxicillin-clavulanic acid (2:1)			-	-	-	-	-	1	3	8	8	14	3	4	1			
Amoxicillin-clavulanic acid (2:1) (UTI)			-	-	-	-	-	-	2	8	3	2	2	-	-			
Imipenem		-	-	-	39	18	1	-	-	1	-	-	-	-				
Cephalothin				-	-	-	-	1	-	-	8	17	8	2	-	23		
Cefotaxime		-	1	17	14	2	1	-	-	1	1	2	2	18				
Cefoperazone				1	4	12	6	1	2	4	2	1	3	23				
Ceftiofur			-	-	2	8	23	2	1	-	-	4	-	-	19			
Florfenicol					-	-	-	-	-	10	35	10	1	1	-	1	1	
Streptomycin						-	-	-	-	13	16	3	2	-	6	7	3	9
Gentamicin					-	1	29	15	3	-	-	1	5	4	1	-	-	
Neomycin					-	1	18	26	4	1	1	-	1	2	5			
Ciprofloxacin	1	17	11	2	2	1	2	-	-	-	4	4	15					
Enrofloxacin	-	6	20	5	1	1	2	1	1	-	-	5	17					
Marbofloxacin	-	5	21	5	2	-	1	2	-	-	7	12	4					
Nalidixic acid				-	-	-	-	4	23	3	2	-	3	-	1	23		
Trimethoprim-sulfamethoxazole (1:19)		-	1	19	9	2	2	2	-	-	-	-	1	23				
Tetracycline					-	-	-	11	16	7	-	-	-	6	14	5	-	
Doxycycline				-	-	-	2	14	13	3	6	6	10	4	1	-		
Colistin			1	-	-	6	51	-	1	-	-	-	-	-	-			

The black areas are the test ranges not included in the test panels for the respective antimicrobial agents. Isolates that had no growth in any of the concentrations were given the lowest MIC value. Isolates with growth in all tested concentrations were given the next serially higher MIC value above the highest tested concentration (white number on black background). The MIC values of amoxicillin-clavulanic acid (2:1) and trimethoprim-sulfamethoxazole (1:19) are expressed as the MIC values of amoxicillin and trimethoprim, respectively. Different gray shading indicates the categories: susceptible—light gray; intermediate—middle gray; resistant—dark gray. For the clinical breakpoints used, please see Materials and Methods, Section 4.2.

**Table 4:** Minimal inhibitory concentrations of the 56 canine and feline *P. aeruginosa* isolates.

Antimicrobial Agent (s)	Number of Isolates for Which the MIC Value (mg/L) Is																	
	0.008	0.015	0.03	0.06	0.12	0.25	0.5	1	2	4	8	16	32	64	128	256	512	1024
Penicillin G		-	-	-	-	-	-	-	-	-	-	-	-	56				
Ampicillin			-	-	-	-	-	-	-	-	-	-	-	-	56			
Amoxicillin-clavulanic acid (2:1)			-	-	-	-	-	-	-	-	-	-	-	-	56			
Imipenem		-	-	-	-	-	8	19	21	5	3	-	-	-				
Cephalothin				-	-	-	-	-	-	-	-	-	-	-	-	56		
Cefotaxime		-	-	-	-	-	-	-	-	-	11	22	16	7				
Cefoperazone				-	-	-	-	1	3	24	20	5	3	-				
Ceftiofur			-	-	-	-	-	-	1	-	-	14	27	11	3			
Florfenicol					-	-	-	-	-	-		-	2	14	28	10	2	
Streptomycin						-	-		-	-	-	2	16	23	9	2	1	3
Gentamicin					-	-	1	10	26	14	4	1	-	-	-	-	-	
Neomycin					-	-	-	-	6	16	12	11	5	5	1			
Ciprofloxacin	-	-	-	6	22	11	7	1	2	3	2	-	2					
Enrofloxacin	-	-	-	-	-	3	21	14	7	3	1	2	5					
Marbofloxacin	-	-		-	-	11	22	8	5	4	1	1	4					
Nalidixic acid				-	-	-	-	-	-	-		-	23	18	3	12		
Trimethoprim-sulfamethoxazole (1:19)		-	-	-	-	-	-	1	3	27	16	5	1	3				
Tetracycline					-	-	-	-	-	1	3	34	12	6	-	-	-	
Doxycycline				-	-		-	-	-	2	6	27	18	3	-	-		
Colistin			-		-	-	3	40	13	-	-	-	-	-	-			

The black areas are the test ranges not included in the test panels for the respective antimicrobial agents. Isolates that had no growth in any of the concentrations were given the lowest MIC value. Isolates with growth in all tested concentrations were given the next serially higher MIC value above the highest tested concentration (white number on black background). The MIC values of amoxicillin-clavulanic acid (2:1) and trimethoprim-sulfamethoxazole (1:19) are expressed as the MIC values of amoxicillin and trimethoprim, respectively. Different gray shading indicates the categories: susceptible—light gray; intermediate—middle gray; resistant—dark gray. For the clinical breakpoints used, please see Materials and Methods, Section 4.2.

**Table 5:** Minimal inhibitory concentrations of the 14 canine and feline *A. baumannii* isolates.

Antimicrobial Agent (s)	Number of Isolates for Which the MIC Value (mg/L) Is																	
	0.008	0.015	0.03	0.06	0.12	0.25	0.5	1	2	4	8	16	32	64	128	256	512	1024
Penicillin G		-	-	-	-	-	-	-	-	-	-	-	7	7				
Ampicillin			-	-	-	-	-	-	-	-	7	3	-	-	4			
Amoxicillin-clavulanic acid (2:1)			-	-	-	-	-	-	-	1	7	2	-	-	4			
Imipenem		-	-	-	2	7	1	-	4	-	-	-	-	-				
Cephalothin				-	-	-	-	-	-	-	-	-	-	-	-	14		
Cefotaxime		-	-	-	-	-	-	-	-	-	9	1	-	4				
Cefoperazone				-	-	-	-	-	-	-	-	1	7	6				
Ceftiofur			-	-	-	-	-	-	-	-	4	4	1	2	3			
Florfenicol					-	-	-	-	-	-	-	-	1	1	11	1	-	
Streptomycin						-	-	-	-	2	1	3	3	1	-	-	-	4
Gentamicin					-	-	4	5	-	1	1	-	-	-	3	-	-	
Neomycin					-	-	4	4	2	-	-	1	2	1	-			
Ciprofloxacin	-	-	-	2	6	1	-	-	-	-	-	-	5					
Enrofloxacin	-	-	7	2	-	-	-	-	-	-	3	2	-					
Marbofloxacin	-	-	2	5	2	-	-	-	-	-	4	1	-					
Nalidixic acid				-	-	-	-	-	4	4	1	-	-	-	-	5		
Trimethoprim-sulfamethoxazole (1:19)		-	-	-	7	3	-	-	-	-	1	-	-	3				
Tetracycline					-	-	2	5	3	-	1	-	-	-	-	3	-	
Doxycycline				2	6	1	2	-	-	-	-	-	3	-	-	-		
Colistin			-	-	-	-	13	1	-	-	-	-	-	-	-			

The black areas are the test ranges not included in the test panels for the respective antimicrobial agents. Isolates that had no growth in any of the concentrations were given the lowest MIC value. Isolates with growth in all tested concentrations were given the next serially higher MIC value above the highest tested concentration (white number on black background). The MIC values of amoxicillin-clavulanic acid (2:1) and trimethoprim-sulfamethoxazole (1:19) are expressed as the MIC values of amoxicillin and trimethoprim, respectively. Different gray shading indicates the categories: susceptible—light gray; intermediate—middle gray; resistant—dark gray. For the clinical breakpoints used, please see Materials and Methods, Section 4.2.

**Table 6:** Origin of the isolates.

Infections	*E. faecalis* (*n* = 49)	*E. faecium* (*n* = 37)	*E. coli* (*n* = 59)	*P. aeruginosa* (*n* = 56)	*A. baumannii* (*n* = 14)
Wound infections					
Dog	16	11	17	12	4
Cat	7	2	5	-	1
Skin infections *					
Dog	10	4	10	34	3
Cat	-	1	-	1	-
Urinary tract infections					
Dog	3	8	3	3	-
Cat	7	2	14	-	-
Respiratory tract infections					
Dog	1	-	1		3
Cat	-	-	-	2	-
Others **					
Dog	5	4	5	4	-
Cat	-	5	4	-	3

* including otitis; ** others include intestinal infections, septicemia, infections of implants, eyes, lymph nodes, inner organs, or joints.

## Data Availability

All data are presented in the text and tables.
